# A Mutation Outside the Dimerization Domain Causing Atypical STING-Associated Vasculopathy With Onset in Infancy

**DOI:** 10.3389/fimmu.2018.01535

**Published:** 2018-07-06

**Authors:** Rohit G. Saldanha, Katherine R. Balka, Sophia Davidson, Brynn K. Wainstein, Melanie Wong, Rebecca Macintosh, Christine K. C. Loo, Martin A. Weber, Vasanth Kamath, CIRCA, AADRY, Fiona Moghaddas, Dominic De Nardo, Paul Edgar Gray, Seth Lucian Masters

**Affiliations:** ^1^Department of Immunology and Allergy, Sydney Children’s Hospital Randwick, Sydney, NSW, Australia; ^2^School of Women and Children’s Health, University of New South Wales, Sydney, NSW, Australia; ^3^Inflammation Division, The Walter and Eliza Hall Institute of Medical Research, Parkville, VIC, Australia; ^4^Department of Medical Biology, The University of Melbourne, Parkville, VIC, Australia; ^5^Children’s Hospital at Westmead, Westmead, NSW, Australia; ^6^Department of Medical Genetics, Sydney Children’s Hospital, Randwick, NSW, Australia; ^7^Department of Anatomical Pathology, NSW Health Pathology, Prince of Wales Hospital, Sydney, NSW, Australia; ^8^School of Medical Sciences, University of New South Wales, Sydney, NSW, Australia; ^9^Clinical Immunogenomics Consortium Australia, Sydney, NSW, Australia; ^10^Australian Autoinflammatory Diseases Registry, Melbourne, VIC, Australia

**Keywords:** stimulator of interferon genes, STING-associated vasculopathy with onset in infancy, interferon, NF-κB, JAK, immunodeficiency

## Abstract

**Background:**

Mutations in the gene encoding stimulator of interferon genes (STING) underlie a type I interferon (IFN) associated disease, STING-associated vasculopathy with onset in infancy (SAVI). Patients suffer cutaneous vasculopathy and interstitial lung disease, but are not known to suffer life-threatening infection.

**Case:**

We describe a child who presented with *Pneumocystis jirovecii* pneumonia in early life, from which he recovered. He went on to suffer failure to thrive, developmental delay, livedo reticularis, and vesicular rash, but without cutaneous vasculitis, and with normal C-reactive protein and erythrocyte sedimentation rates. At 3 years of age, he developed life-threatening pulmonary hypertension.

**Methods:**

Whole genome sequencing (WGS) was performed using the Illumina HiSeqX10 platform and the Seave platform was used for bioinformatic analysis. mRNA expression of IFN-stimulated genes and inflammatory cytokines from peripheral blood mononuclear cells was determined by quantitative polymerase chain reaction. Luciferase assay was used to model IFNβ and NF-κB activity *in vitro*.

**Results:**

WGS revealed a *de novo* mutation p.Arg284Ser in STING at an amino acid previously associated with SAVI. Although this mutation did not fall in the dimerization domain (DD), mRNA analysis revealed constitutive IFN-gene activation consistent with an interferonopathy, which correlated to STING activation *in vitro*. The patient was treated with corticosteroids and the JAK inhibitor Ruxolitinib, resulting in a rapid improvement of pulmonary hypertension, general well-being, and resolution of the IFN gene signature. However, he did go on to evolve a nasal septal erosion suggesting incomplete control of disease.

**Conclusion:**

This case provides molecular evidence to support the p.Arg284Ser variant in STING exerting pathogenicity through a gain-of-function mechanism. The lack of cutaneous vasculitis or elevated systemic inflammatory markers, and the occurrence of an opportunistic infection are notable, and raise the possibility that variants outside the STING DD may potentially manifest with an atypical SAVI phenotype. Nevertheless, there was an objective clinical improvement in response to JAK inhibition.

## Introduction

Type I interferons (IFNs α and β) play a crucial role in immune mediated protection from viral infection. Stimulator of interferon genes (STING), encoded by the gene *TMEM173*, is a key intermediary in activating this type I IFN response (1). Once activated, STING induces the phosphorylation of transcription factors IRF3 and NF-κB, promoting their nuclear translocation and the transcription of IFNs and inflammatory cytokines, respectively. Type I IFNs act *via* the IFN α/β receptor (IFNAR), which in turn activates a number of down-stream signaling pathways, most notably the JAK–STAT pathway. IFNAR agonism leads to the transcriptional regulation of over 2,000 genes, referred to as IFN-stimulated genes (ISGs). Heterozygous gain-of-function (GoF) mutations in *TMEM173* result in constitutive activation of STING and the clinical syndrome known as STING-associated vasculopathy with onset in infancy (SAVI) (2). To date, a total of seven GoF mutations have been reported to induce SAVI, a disease characterized by neonatal–onset inflammation with raised inflammatory markers, cutaneous vasculopathy, and interstitial lung disease (2–9). SAVI has been classified an as interferonopathy as it is characterized by elevated levels of type I IFNs and ISGs. Activation of STING also triggers NF-κB, and mouse models suggest that this could contribute to the pathogenesis of SAVI (10). Typically, activation of NF-κB is associated with increased C reactive protein (CRP) in patients with inflammation, and CRP is also elevated in SAVI.

## Case

**Figure 1:**
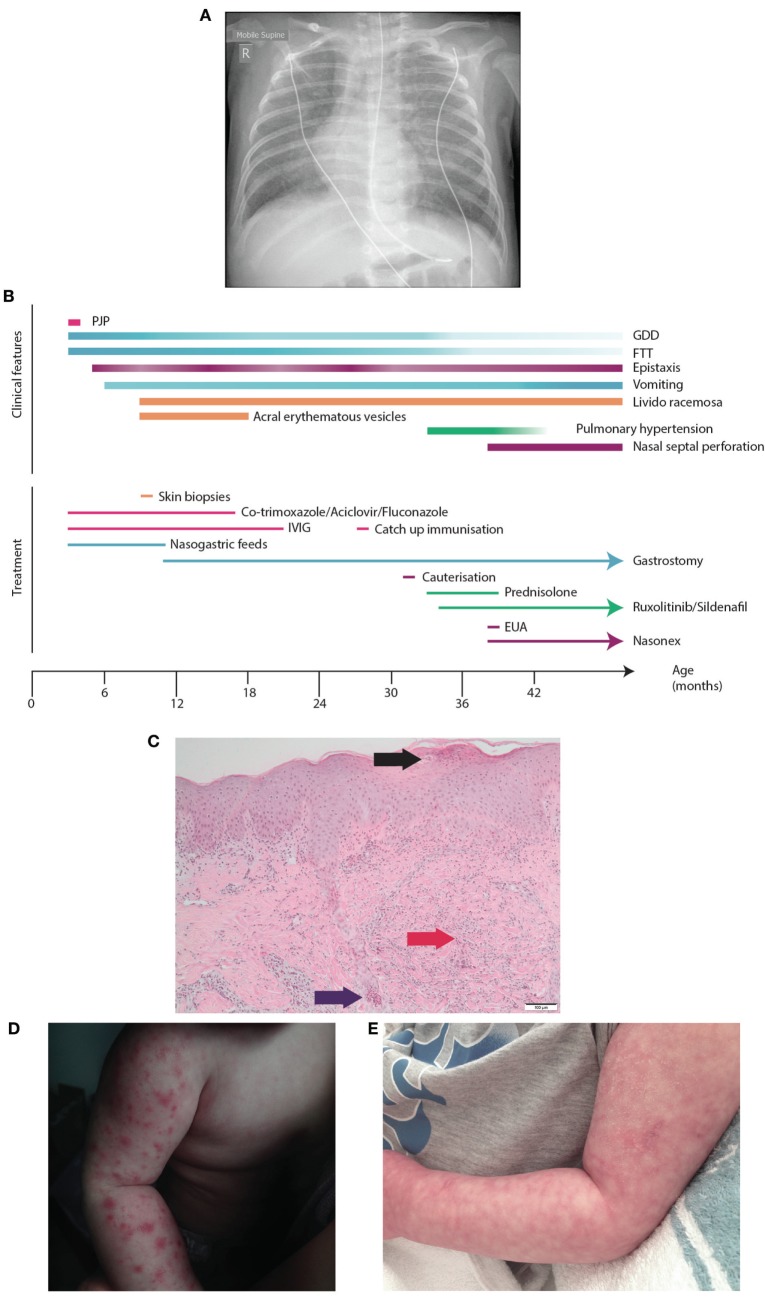
Presentation of SAVI R284S. **(A)** Chest X-ray of patient demonstrating diffuse hazy changes present bilaterally in the lung fields. The X-ray appearance was consistent with an interstitial pneumonitis which was initially attributed to his *Pneumocystis jirovecci* infection requiring non-invasive ventilator support and then an escalation to intubation and ventilator support. **(B)** Time-line for disease manifestation and treatments undergone by the index case. **(C)** Histopathology of the vesicles from the skin biopsy of the arm demonstrating a pustule (black arrow) with dermal neutrophilia (red arrow) and associated leukocytoclasis. Neutrophils are also present within the lumen of a sweat duct (blue arrow). **(D)** Photograph of the acral erythromatous papules and vesicles that were present on both upper and lower limbs which resolved into **(E)** a livedo racemosa pattern.

A 3-year-old boy born to non-consanguineous parents of Chinese/Malaysian ethnicity presented at 2 months of age with acute respiratory distress requiring mechanical ventilation and an interstitial pneumonitis on X-ray (Figure 1A). Bronchoalveolar lavage identified *Pneumocystis jirovecii* by both PCR and immunofluorescence. He was treated with co-trimoxazole and made a full recovery (Figure 1B). Immunological work up performed at the time revealed normal immunoglobulin levels, with CD4+ and CD8+ lymphopenia (Table 1). The CD8+ count normalized rapidly but his CD4+ lymphopenia persisted until he was 5 months of age. CD19+ B cell and NK cell numbers were normal; however, the B cell numbers climbed and have remained high (range 2.5–6.0 × 10^9^/L—reference range 0.2–2.1 × 10^9^/L). T cell immunophenotyping identified no abnormality (Table 1). Mitogen-specific T-cell blastogenesis with phytohemagglutinin (PHA) was preserved; however, T-cell stimulation with anti-CD3 in early life was absent, and stimulation with anti-CD3/anti-CD28 at 9 months of age was reduced threefold compared with a control sample (Table 1). He displayed features of global developmental delay at 7 months and was noted to be hypertonic floppy with normal deep tendon reflexes and a normal MRI of the brain. These features improved slowly and required intensive allied health (physiotherapy, occupational therapy, and dietician) support that is ongoing. He had failure to thrive complicated by frequent vomiting necessitating insertion of a gastrostomy at 10 months of age. Endoscopy revealed a thickened (but non-occlusive) pyloric antrum. He developed acral erythematous papules and vesicles on his upper and lower limbs. Skin biopsy revealed superficial subcorneal neutrophil microabscess formation with superficial and deep dermal and subcutaneous neutrophilia (Figure 1C). Neutrophils were also present within the lumen of a sweat duct. There was no evidence of vasculitis, although interstitial leukocytoclasis was present. The acral papules (Figure 1D) gradually resolved around 9 months while the skin of his extremities demonstrated a livedo racemosa pattern (Figure 1E). Notably, at the time of presentation he had raised inflammatory markers; however, these decreased to within normal range when tested during a routine follow-up (CRP < 3 mg/L, erythrocyte sedimentation rate < 14 mm/h) (Table 1).

**Table 1:** Summary of immunological investigations.

	2 Months	37 Months	NR
**Inflammatory markers**			
CRP	25 mg/L	<1 mg/L	<3 mg/L
ESR	34 mm/h	11 mm/h	0–14 mm/h
Ferritin	360 µg/L	Not performed	20–300 µg/L
**Immunoglobulins**			
IgG	11.1 g/L	10.10 g/L	4.31–11.09 g/L
IgA	1.09 g/L	1.12 g/L	0.15–1.42 g/L
IgM	2.39 g/L	0.62 g/L	0.42–1.61 g/L
IgE	45 IU/L	N/A	0–60 IU/L
**Immunophenotyping**			
CD45+	2.6 × 10^9^/L *(NR: 3.8–7.6)*	6.6 × 10^9^/L *(NR: 2.2*–*6.6)*	
CD3+	1.35 × 10^9^/L *(NR: 2.3*–*6.5)*	3.76 × 10^9^/L *(NR: 0.9*–*4.5)*	
CD4+	0.94 × 10^9^/L *(NR: 1.5*–*5.0)*	1.98 × 10^9^/L *(NR: 0.5*–*2.4)*	
CD8+	0.42 × 10^9^/L *(NR: 0.5*–*1.6)*	1.65 × 10^9^/L *(NR: 0.3*–*1.6)*	
CD19+	1.07 × 10^9^/L *(NR: 0.6*–*3.0)*	2.51 × 10^9^/L *(NR: 0.2*–*2.1)*	
CD16+/56+	0.16 × 10^9^/L *(NR: 0.1*–*1.3)*	0.33 × 10^9^/L *(NR: 0.1*–*1.0)*	
**Lymphocyte proliferation studies (stimulation index) at 9 months of age**			
PHA	39.07 (control = 32; NR > 17.2)	
Anti-CD3+	3.36 (control = 13.74; NR > 2.3)	
Anti-CD3+/anti-CD28+	9.14 (control 28.04; NR > 11.2)	

*Immune phenotyping was performed at 2 months of age when the patient presented with Pneumocystis jirovecii pneumonia and more recently at 37 months of age during a routine follow-up. Lymphocyte proliferation performed at 9 months shows an index ratio of mean counts per minute with antigen stimulation per mean count per minute without antigen stimulation*.

*CRP, C-reactive protein; ESR, erythrocyte sedimentation rate; NR, normal range; PHA, phytohemagglutinin*.

The patient was managed as a primary immunodeficiency with prophylactic co-trimoxazole and immunoglobulin replacement. He did not suffer from any further significant infections. Co-trimoxazole was ceased at 2 years of age and immunoglobulin replacement ceased at 21 months of age, since when he has remained free of infections. The patient underwent routine immunization and demonstrated adequate post vaccination serological responses.

At 33 months, the patient developed a pulmonary hypertensive crisis episode characterized by hypoxia following minor stimulation. Electrocardiogram suggested right ventricular (RV) hypertrophy, subsequently confirmed by echocardiogram which demonstrated suprasystemic pulmonary hypertension and a pressure loaded RV. Notably cardiac echocardiogram performed at age 21 months was normal. High resolution computed tomography of the chest to identify a pulmonary cause was abandoned due to hypertensive crises on stimulation. He was subsequently started on the pulmonary specific vasodilator, sildenafil.

**Figure 2:**
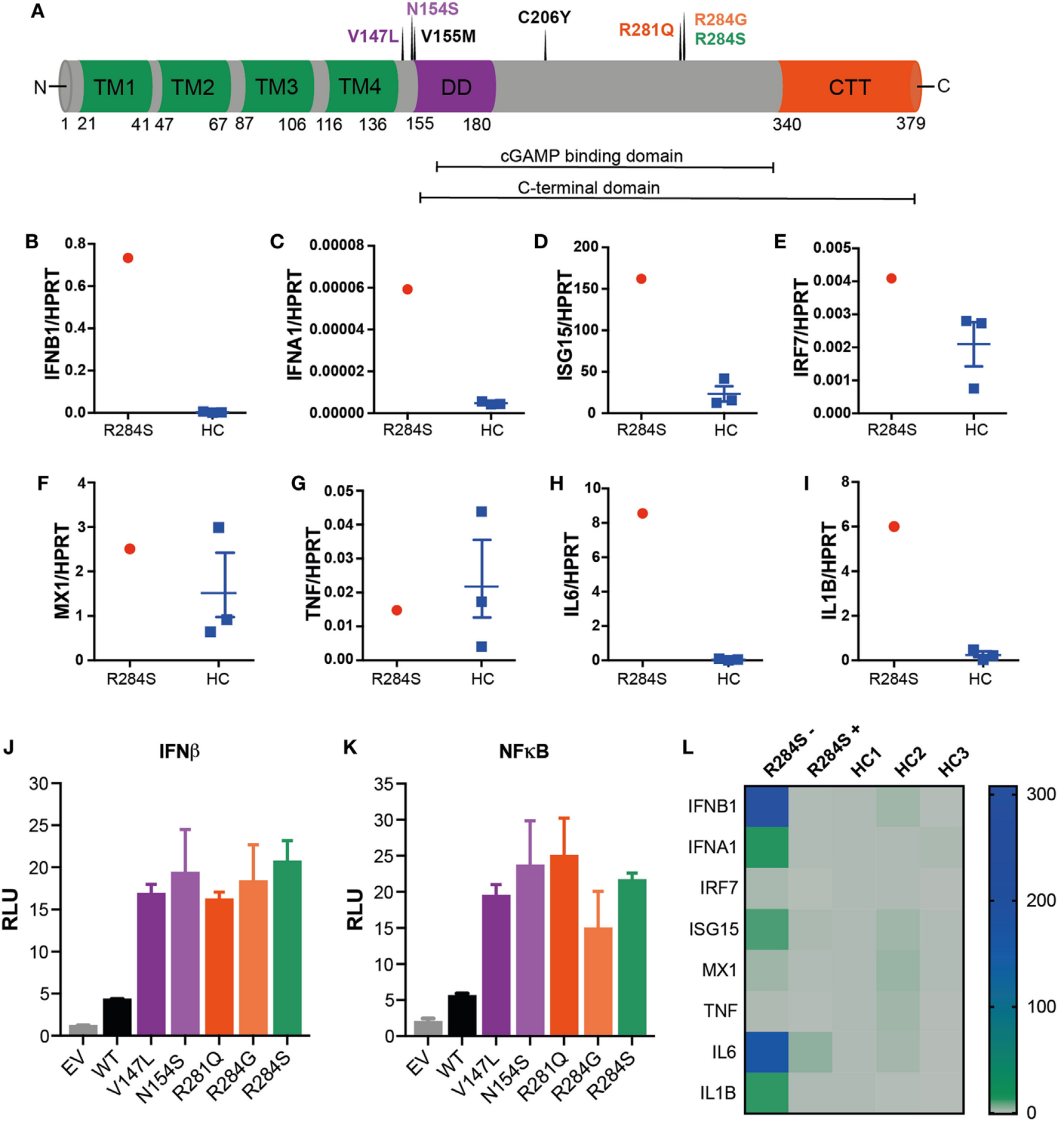
R284S is a pathogenic gain-of-function stimulator of interferon genes (STING) mutation. **(A)** Schematic structure of human STING: four transmembrane domains (TM, green), the dimerization domain (DD, purple), the C-terminal tail (CTT, red) region, and indicating the locations of known STING-associated vasculopathy with onset in infancy (SAVI) mutations. **(B–I)** Peripheral blood mononuclear cells (PBMCs) were isolated from the patient and three healthy controls (HC) and analyzed *ex vivo* for expression of inflammatory mediators by quantitative polymerase chain reaction (qPCR) analysis. Data for HC are shown as mean ± SEM of the three individuals. **(J)** IFNβ and **(K)** NF-κB luciferase reporter activity was monitored following transfection of HEK293T cells with an empty vector (EV) control, wild-type (WT) STING, or the indicated SAVI mutants of STING. Firefly luciferase reporter activity was normalized to the renilla control and presented as relative luciferase units (RLU). Data are representative of four independent experiments and shown as mean ± SD of three technical replicates. **(L)** Heatmap showing the mRNA expression of indicated inflammatory mediators as measured by qPCR analysis of PBMCs from the patient R284S before (−) or after (+) treatment with JAK inhibitor Ruxolitinib. This is compared to three HCs, with data shown relative to HC1.

To establish if there was a genetic cause of this disease, whole genome sequencing (WGS) was performed on the family trio. This identified a novel *de novo* missense variant c.852G>T in exon 7 of 8 in the gene *TMEM173*, encoding p.Arg284Ser in the protein STING (Figure 2A). The variant was confirmed by Sanger sequencing, and is not present in the Exac database (11). *In silico* analysis predicted the mutation to be pathogenic based on; the physiochemical difference between arginine and serine (Grantham score 110); SIFT prediction (deleterious), PolyPhen (probably damaging), CADD (scaled = 26.7), mutation taster (disease causing), and Provean (deleterious). Interestingly, a previous case report documented a different *de novo* change in the same amino acid residue; c850A>G (pArg284Gly, R284G) in a patient suffering from SAVI with upregulation of IFN genes secondary to constitutive STING activation (8).

We hypothesized the substitution of a serine for an arginine at position 284 in *TMEM173* would result in constitutive activation of STING and consequently, autoinflammatory disease charactered by IFNαβ and NF-κB activation. To investigate this, peripheral blood mononuclear cells (PBMCs) were isolated from patient blood samples and analyzed for mRNA expression of ISGs and inflammatory cytokines by quantitative polymerase chain reaction (qPCR). Compared to healthy controls (HCs) our patient showed an IFN-gene signature with markedly raised levels of IFNB1 and the ISGs IFNA1, ISG15, IRF7, and MX1 supporting the diagnosis of an interferonopathy (Figures 2B–F). We also interrogated the mRNA expression of several NF-κB-dependent inflammatory cytokines: TNF, IL6, and IL1B. Interestingly, while TNF was not elevated, IL6 and IL1B were significantly upregulated (Figures 2G–I).

We next sought to directly determine if R284S triggers STING activation. STING expression plasmids encoding R284S and other known SAVI mutations (Figure 2A) were created by site-directed mutagenesis for *in vitro* studies. We then transfected HEK293T cells with wild-type (WT) and mutant versions of STING before assessing IFNβ and NF-κB activation using luciferase reporter systems. The R284S mutant STING induced high IFNβ activity compared to WT STING, similar to the previously published STING GoF mutants (Figure 2J). We further found that all SAVI mutants assayed, including R284S, induced markedly enhanced NF-κB activation compared to WT STING (Figure 2K). Therefore, *in vitro* analysis confirms R284S as a pathogenic GoF STING mutation.

After confirming the diagnosis of an interferonopathy, the patient was treated with a combination of oral prednisolone (2 mg/kg/day) and the JAK 1/2 inhibitor Ruxolitinib (5 mg daily) (12) which was well tolerated. His mobility and demeanor improved within days of steroid therapy including quality of sleep, respiratory effort, and cessation of his frequent vomiting episodes. His appetite improved, with weight gain of approximately 1.5 kg in 4 weeks. Serial weight velocity has subsequently increased from <3rd to 20th percentile in 5 months. His pulmonary pressures became sub-systemic within 8 weeks of sildenafil, steroids, and Ruxolitinib therapy. Initial steroid therapy coincided with a notable neurodevelopmental improvement. He made rapid gains in mobility (gross motor), self-feeding (fine motor), sleep/social interactions (personal social), and receptive speech (communication) compared to previous years while on intensive support. This correlated with a reduction in ISGs and inflammatory cytokines in the patient PBMCs following 6 months of treatment (Figure 2L). However, the lividinous rash persisted, and 6 months later the patient developed complete loss of his nasal septum while continuing on Ruxolitinib therapy. There was partial recrudescence of his gastrostomy feed related vomiting when his steroids were weaned. In summary, the treatment regime resulted in an excellent systemic response; however, it is possible that some tissue specific processes may remain uncontrolled.

## Discussion

The clinical phenotype in almost all SAVI patients has included persistent systemic inflammation, cutaneous rash, failure to thrive, vasculitis of varying degrees, and interstitial lung disease (2–9). There are now two reports of pulmonary arterial hypertension in SAVI, presumed secondary to interstitial lung disease (4, 9). This may be the case in our patient, where a full work-up of the chest was not possible. However, our patient displays atypical features for SAVI, with no evidence of vasculitis or ongoing systemic inflammation, an atypical skin rash, and presentation with opportunistic infection, which suggested primary immunodeficiency. A different mutation at the same site as our patient, reported by Melki et al. (R284G) had interstitial lung disease but no significant inflammation, leading to suggestions of phenotypic differences between the two main sites of GoF mutations in STING (8).

Even more salient to the current case, while this manuscript was in preparation, Konno et al. described a 9-month-old infant with the same *de novo* heterozygous mutation in STING (p.R284S) (13). That patient presented without typical features of SAVI, but with a severe neck abscess, and later succumbed to pneumonia. *In vitro* studies conducted by Konno et al. agree with the data presented here that the p.R284S STING mutation is constitutively active. Significantly, we were able to confirm this finding through *ex vivo* analysis of patient cells. R284 is located within the cyclic dinucleotide binding domain of STING; however, its mutation does not seem to alter ligand affinity (8, 13). An alternate explanation could be that R284S changes/blocks the ubiquitination of STING at K289, which may increase its activation when stimulated (14), or that this region is required for appropriate shuttling of STING between the endoplasmic reticulum and Golgi (15). Overall, our study and that of Konno et al. provide more evidence that mutations outside the dimerization interface still cause GoF SAVI, but may present with atypical disease symptoms. A summary and comparison of the findings from all three patients with heterozygous GoF mutations in STING R284 is presented in Table 2.

**Table 2:** Comparison of phenotypic changes associated with heterozygous mutation at p.R284 stimulator of interferon genes reported in the literature and this study.

	R284G (8)	R284S (13)	R284S
Phenotype			
Sex	F	M	M

Age	25 years (*alive*)	9 months (*deceased)*	3 years (*alive*)

Cutaneous lesions	Livido	NIL	Livido
	Acrocyanosis		Acral neutrophilic dermatosis
	Palatal/nasal septum necrosis		Nasal septum necrosis

Lungs	Interstitial lung disease (ILD)	ND	Pulmonary hypertension

Infection	Recurrent bacterial infections in early life	Neck abscess	PJP infection at 2 months of life
		Fatal pneumonia	

Elevated CRP/ESR	NIL	NIL	NIL

Immune perturbations	ND	ND	Reduced CD3/CD28 lymphocyte proliferation

**Transcription factor activation and binding**			
IRF3 phosphorylation	+++ (HEK293T cells)	+++ (MEF cells^a^)	ND

IFNβ reporter activity	++ (HEK293T cells)	+++ (HEK293T cells)	++ (HEK293T cells)

NF-κB reporter activity	ND	+++ (HEK293T cells)	++ (HEK293T cells)

STAT1 phosphorylation	+++ (*ex vivo* PBMCs)	+++ (MEF cells^a^)	ND

**Cytokine expression**			
Type 1 IFN	ND	− (MEF cells^a^)	+++ (*ex vivo* PBMCs)

IL1	ND	ND	+++ (*ex vivo* PBMCs)

IL6	ND	++ (serum)	+++ (*ex vivo* PBMCs)

TNF	ND	ND	− (*ex vivo* PBMCs)

**Response to treatment with JAK inhibitor**			
*In vitro* assay	+ve (ruxolitinib)	+ve (tofacitinib)	ND

*Ex vivo* assay	ND	ND	+ve (ruxolitinib)

*In vivo* treatment	NIL	Deceased	Yes (ruxolitinib)

*ILD, interstitial lung disease; MEF, mouse embryonic fibroblasts; ND, not done; PBMCs, peripheral blood mononuclear cells; PJP, Pneumocystis jirovecii pneumonia; ESR, erythrocyte sedimentation rate; CRP, C reactive protein*.

*^a^In vitro reconstitution of TMEM173^−/−^ MEF cells*.

Infections have not been a predominant part of SAVI, although patients may have recurrent bacterial upper respiratory infections (including pseudomonas infections), which is likely to be attributable to structural predisposition to infection from evolving interstitial lung disease rather than an immune defect (2–9). Patients have also demonstrated lymphopenia, a feature seen in the first STING GoF (N153S) mutant mouse (10). Recently, Bouis et al. characterized another heterozygous STING mutation in mice (V154M) which demonstrated a severe combined immunodeficiency phenotype affecting T/B/NK cell numbers (16). This was associated with an intrinsic defect in anti-CD3/CD28 T cell proliferation that was partially IFN dependent and correlates with our phenotypic observation of reduced anti-CD3/CD8 stimulated cell proliferation and immunodeficiency in our patient. From this perspective, it is noteworthy that our patient demonstrated a poor response to anti-CD3 but not to PHA stimulation in early life. An intriguing feature of our patient is that he never relapsed with *Pneumocystis jirovecii* pneumonia (PJP) after coming off co-trimoxazole, which would be unusual for a patient with a primary immunodeficiency predisposing to this organism, such as CD40L deficiency. Therefore, perhaps this predisposition reduced over time. In terms of a link between STING and PJP predisposition, type I IFNs are thought to play a role in the control of this fungus (17); however, linking constitutive STING activation to *Pneumocystis* infection remains speculative.

The STING N153S mutant mouse closely resembles some manifestations of SAVI such as splenomegaly, pleural effusions, respiratory distress, and ulcerative skin lesions (10). Interestingly, when crossed to mice deficient in IRF3 (preventing the production of IFNβ via STING) the mice still developed dysregulation of immune cells and lung disease. Therefore, NF-κB-dependent cytokines may play a dominant role in the phenotype observed for this SAVI mouse model. However, while the N153S mice developed chronic perivascular inflammation and organized thrombosis in pulmonary blood vessels, they did not develop the pulmonary fibrosis observed in SAVI and demonstrated a milder upregulation of the type I IFN signature compared to their human counterparts. This provides an interesting comparison to the atypical features observed in our patient with the R284S mutation. While the acute pulmonary hypertensive crisis was attributed to interstitial lung disease common to SAVI cases, the patient did not demonstrate clinical signs of lung parenchymal disease (tachypnea or hypoxemia). Moreover, his overnight pulse oximetry studies revealed a normal oxygen saturation range >92% in ambient room air. We surmise that the pulmonary hypertension in the absence of clinically evident lung disease might represent a primary vascular pathological process similar to that seen in the SAVI mouse model. Whether mutations in R284 can result in preferential, site-specific vasculitis given the absence of cutaneous vasculitis in our patient is unknown. It would be very interesting to know if a mouse model encoding a mutation analogous to R284S could reveal a different phenotype and help to explain why mutations outside the dimerization domain of human STING display features atypical of SAVI.

This case reinforces the atypical presentation of two previous patients with mutations at position R284 and raises the possibility that variants at this locus may present a different phenotype to patients with variants elsewhere in the STING protein. This is the first patient with a STING GoF mutation documented to suffer from PJP infection, which is an important consideration for SAVI patients who display lymphopenia and impaired T-cell proliferation. *In vitro* functional analysis of the R284S STING mutation found evidence of IFN and NF-κB activation, however, did not reveal a basis for potential genotype–phenotype correlation. Our study supports the benefit of JAK inhibition with Ruxolitinib for these patients, but raises the possibility that this intervention may provide incomplete control of some symptoms.

## Methods

### Patient and Study Approval

Written informed consent for publication of this case report was obtained from the patients guardians. The patient was consented for WGS under South East Sydney Local Health District ethics (HREC/11/POWH/152). The patient was recruited to the Australia Autoinflammatory Diseases Registry (AADRY, HREC/15/Mon/31) for *ex vivo* examination of PBMCs. HC PBMC were obtained from blood collected at the Volunteer Blood Donor Registry under Walter and Eliza Hall Institute of Medical Research HREC 10/02.

### Whole Genome Sequencing

Whole genome sequencing was performed using the Illumina HiSeqX10 platform, and the Seave platform was used for bioinformatic analysis.

### Lymphocyte Functional Assays

Anti-CD3, anti-CD28-, and PHA-stimulated lymphocyte proliferation assays were performed on whole blood, using uptake of tritiated thymidine, as a clinical test through SA Pathology, Adelaide, South Australia.

### PBMC Isolation and mRNA Expression Analysis

Human PBMCs were purified from whole blood over a Ficoll density gradient (GE Healthcare) and lysed for RNA purification using RNeasy Plus Mini Kit (Qiagen) as directed by the manufacturer. RNA was reverse transcribed to cDNA using SuperScript III Reverse transcriptase (Thermo Fisher). qPCR was performed with Maxima SYBR Green master mix (Thermo Fisher) using primers designed for the specified genes. Expression of target genes was normalized to the expression of the housekeeping gene *HPRT*. Primer sequences used can be provided upon request.

### Generation of STING Variants

To generate plasmids expressing STING carrying the SAVI mutations a human STING cDNA template (pEF-BOS-mCitrine-hSTING) was mutated using the QuikChange Lightning Mutagenesis Kit (Agilent Technologies). The following mutagenesis primers were used:

V147L, 5′-GCTGAGATCTCTGCA****T**TG**TGTGAAAAAGGG-3′;
N154S, 5′-GAAAAA GGGAATTTC**A**G**C**GTGGCCCATGGG-3′;
R281Q, 5′-GCTGGCTTTAGC**C**A**G** GAGGATAGGCTTGAGCAGGC-3′;
R284G; 5′-GGCTTTAGCCGGGAGGAT****G**GG**CTTGAGCAGGCC-3′;
R284S, 5′-GGCTTTAGCCGGGAGGAT **AG**C**** CTTGAGCAGGCC-3′.

### Luciferase Assay

Approximately 1.5 × 10^5^ HEK293T cells were seeded per well in a 24-well tissue culture plate. In each well 500 ng of expression plasmids were transiently transfected per well along with 100 ng of either an IFNβ or NF-κB firefly luciferase reporter plasmid and 10 ng renilla plasmid as an internal control. After 24 h, cells were lysed with Passive Lysis Buffer (Promega), and luminescence was measured on a luminometer using the Dual-Luciferase Reporter Assay System substrates (Promega). Conditions were performed in technical triplicates and firefly luciferase values were normalized to the renilla control.

## Ethics Statement

The patient was consented for whole genome sequencing (WGS) under South East Sydney Local Health District ethics (HREC/11/POWH/152). The patient was recruited to the Australia Autoinflammatory Diseases Registry (AADRY, HREC/15/Mon/31) for *ex vivo* examination of PBMCs. Healthy control PBMC were obtained from blood collected at the Volunteer Blood Donor Registry under Walter and Eliza Hall Institute of Medical Research HREC 10/02.

## Author Contributions

RS, BW, MW, RM, CL, MAW, VK, and PG were involved in collecting phenotypic data from the patient. KB, SD, FM, DD, and SM performed or analyzed *ex vivo* and *in vitro* experiments. CIRCA managed genomic sequencing. AADRY managed *ex vivo* analysis. RS, KB, SD, FM, DD, PG, and SM wrote the manuscript.

## Conflict of Interest Statement

The authors declare that the research was conducted in the absence of any commercial or financial relationships that could be construed as a potential conflict of interest.
